# Effects of water availability and pest pressures on tea (*Camellia sinensis*) growth and functional quality

**DOI:** 10.1093/aobpla/plt054

**Published:** 2013-12-02

**Authors:** Selena Ahmed, Colin M. Orians, Timothy S. Griffin, Sarabeth Buckley, Uchenna Unachukwu, Anne Elise Stratton, John Richard Stepp, Albert Robbat, Sean Cash, Edward J. Kennelly

**Affiliations:** 1 Sustainable Food and Bioenergy Systems Program, Department of Health and Human Development, Montana State University, Bozeman, MT 59715, USA; 2 Department of Biology, Tufts University, Medford, MA 02155, USA; 3 Friedman School of Nutrition Science and Policy, Tufts University, Boston, MA 02111, USA; 4 Department of Earth Sciences, Boston University, Boston, MA 02215, USA; 5 Department of Biochemistry, The Graduate Center of the City University of New York, New York, NY 10016, USA; 6 Department of Anthropology, University of Gainesville, Gainesville, FL 32611, USA; 7 Department of Chemistry, Tufts University, Medford, MA 02155, USA; 8 Department of Biological Sciences, Lehman College, Bronx, NY 10468, USA

**Keywords:** *Camellia sinensis*, catechins, climate change, herbivory, methylxanthines, precipitation, tea, total phenolic concentrations.

## Abstract

Extreme shifts in water availability linked to global climate change are impacting crops worldwide. The present study examines the direct and interactive effects of water availability and pest pressures on tea (*Camellia sinensis*; Theaceae) growth and functional quality. Manipulative greenhouse experiments were used to measure the effects of variable water availability and pest pressures simulated by jasmonic acid (JA) on tea leaf growth and secondary metabolites that determine tea quality. Water treatments were simulated to replicate ideal tea growing conditions and extreme precipitation events in tropical southwestern China, a major centre of tea production. Results show that higher water availability and JA significantly increased the growth of new leaves while their interactive effect was not significant. The effect of water availability and JA on tea quality varied with individual secondary metabolites. Higher water availability significantly increased total methylxanthine concentrations of tea leaves but there was no significant effect of JA treatments or the interaction of water and JA. Water availability, JA treatments or their interactive effects had no effect on the concentrations of epigallocatechin 3-gallate. In contrast, increased water availability resulted in significantly lower concentrations of epicatechin 3-gallate but the effect of JA and the interactive effects of water and JA were not significant. Lastly, higher water availability resulted in significantly higher total phenolic concentrations but there was no significant impact of JA and their interaction. These findings point to the fascinating dynamics of climate change effects on tea plants with offsetting interactions between precipitation and pest pressures within agro-ecosystems, and the need for future climate studies to examine interactive biotic and abiotic effects.

## Abstract (precis)

Extreme shifts in water availability linked to global climate change are impacting crops worldwide. This study examines effects of water availability and pest pressures on the growth and functional quality of tea, the world's most consumed beverage after water. Results show that higher water availability and pest pressures significantly increased the growth of new leaves while their effect on tea quality varied with individual secondary metabolites. Findings point to the fascinating dynamics of climate change effects on tea plants with offsetting interactions between rainfall and pest pressures and the need for future climate studies to examine interactive environmental effects.

## Introduction

Crops around the world are being impacted by extreme shifts in water availability linked to global climate change. For example, droughts and floods are reducing the yields of many crops (Porter and Semenov 2005; Lobell *et al.* 2011) as well as altering their quality (Coley 1998; Jamieson *et al.* 2012). In fact, precipitation is the most important climatic determinant, along with temperature, for plant growth and survival (Boisvenue and Running 2006). Future climatic projections show strong precipitation heterogeneity depending on geographic location, including an increase in the number of heavy precipitation events as well as longer and more intense droughts (Orlowsky and Seneviratne 2012; Seneviratne *et al.* 2012). Crop performance is further impacted by indirect climatic influences via alterations in ecological interactions such as pest pressures (Berggren *et al.* 2009; Brenes-Arguedas *et al.* 2009; Schepp 2009). Although the magnitude and direction of future climatic-induced alterations to water availability remain uncertain, it is recognized that these changes will be notable and often exceed plant adaptive capacity (IPCC 2007).

Given present and future water availability scenarios, research is needed to understand crop responses to both direct and indirect effects of climate change for future food security. While previous research has documented the impact of extreme precipitation events on crop yields (Ewert *et al.* 2005; Porter and Semenov 2005; Nelson *et al.* 2009; Schlenker and Lobell 2010; Lobell *et al.* 2011), less is known about the direct and interactive effects of water availability and pest pressures on crop quality. Crop quality is largely determined by nutrient and secondary metabolite profiles via their effects on functional and sensory characteristics for human consumers. Secondary metabolites serve as defence compounds in plants that vary in concentration with a range of environmental, genetic and management conditions, including water availability and pest pressures (Herms and Mattson 1992; Glynn *et al.* 2007; Gutbrodt *et al.* 2011, 2012; Tharayil *et al.* 2011; Atkinson and Urwin 2012; Kruidhof *et al.* 2012; Ahmed *et al.* 2013). Changes induced by both water availability and pest pressures are mediated via signalling pathways (Atkinson and Urwin 2012) that can cause an increase or decrease in the concentrations of secondary metabolites (Gutbrodt *et al.* 2011; Kruidhof *et al.* 2012).

The present study examines the direct and interactive effects of water availability and pest pressures on the functional quality of tea (*Camellia sinensis*; Theaceae). Tea plants, the source of the world's most widely consumed beverage after water, are geographically located in high-risk regions for climate change. Our preliminary work has suggested that tea functional quality drops significantly with extreme precipitation events that accompany the annual onset of the East Asian monsoon and that monsoon patterns are shifting. Tea functional quality is largely determined by polyphenolic catechin and methylxanthine secondary metabolites that are responsible for its antioxidant, anti-inflammatory, cardioprotective and stimulant properties for human consumers (Lin *et al.* 2003). Catechins and methylxanthines are found in the highest concentrations in young expanding leaves, those harvested for commercial tea, and human consumers are able to perceive changes in the concentrations of these metabolites by their bitterness, astringency and sweet aftertaste (Ahmed *et al.* 2010). Since the concentrations of these compounds are predicted to increase following herbivory, increasing pest pressures during the rainy season (Coley 1998) could offset the effects of heavy rainfall.

In this study, manipulative greenhouse experiments were used to measure the effects of variable water availability and pest pressures on secondary metabolites that determine tea quality. Water treatments were simulated to replicate ideal tea growing conditions and extreme precipitation events in tropical southwestern China, a major centre of tea production located in a high-risk region for climate change (Maplecroft 2011). Pest pressures were experimentally simulated here through the application of the plant hormone jasmonic acid (JA) to young tea leaves (McDowell and Dangl 2000; Kruidhof *et al.* 2012). It is well known that an increase in water availability can cause an increase in growth and a decline in secondary metabolites (Brenes-Arguedas *et al.* 2006); whether simulated pest pressures would counter this response is unknown. We hypothesized that increased water availability would indeed lead to lower concentrations of tea secondary metabolites, but that simulated pest pressures would offset these direct effects of water availability.

## Methods

### Plant material

Tea plants (*C. sinensis*; Theaceae) of ∼2 years of age were purchased from Logee's Greenhouse (Danielson, CT, USA). Plants were transplanted into 6-inch plastic pots (total volume 1800 mL) with four drainage holes at the base of each pot. A total of 1300 mL of soil mix that comprised 50 % pearlite and 50 % peat moss was added to each pot. The soil mixture was selected to facilitate quick drainage. Plants were fertilized (Osmocote^©^ Plus 15-9-12, Marysville, OH, USA) 1 week prior to the experimental period. A total of 120 tea plants were included in the experiment.

### Greenhouse set-up

Tea plants were maintained and treated at the greenhouse facility of the Weld Hill Research Building at the Arnold Arboretum, Harvard University (Jamaica Plain, MA, USA). One greenhouse room was used for the present experiment. Temperature, humidity and shade conditions were selected to reflect ideal tea growing conditions. The temperature was maintained at a range of 20–22 °C with a humidity range of 60–70 % and steady air circulation. Shade was set at 50 % over-storey density. Plants were randomly assigned to each water availability and pest pressure treatment and were labelled with treatment identifiers. Tea plants were moved on a weekly basis to eliminate any possible location effects within the greenhouse.

### Water availability treatments

Water availability treatments involved altering the soil moisture content of tea plants to simulate conditions that exist during the spring harvest in tropical southwestern China and extreme precipitation events of drought and heavy monsoon rains (Dou *et al.* 2007), hereafter termed moderate water, low water and high water, respectively. A total of 120 tea plants were treated under each of the three water availability treatments (40 tea plants per treatment) on the basis of field capacity of the experimental soil mixture (32 %) as well as soil moisture of field conditions at the reference location in southwestern China during mean and extreme precipitation levels. The moderate-water treatment was maintained at 12–16 % soil moisture content with drainage, the low-water treatment was maintained at 4–8 % soil moisture content with drainage and the high-water treatment was maintained at 28–32 % soil moisture content with no drainage. Water treatments were applied for 6 weeks before experimental harvest to quantify leaf secondary metabolites.

### Simulated pest pressure treatments with JA

The application of JA to tea leaves was used to simulate pest pressure on the basis of previous studies that have shown JA application to produce induced resistance, marked by an upregulation of secondary metabolic activity that simulates plant response by actual herbivory leaves (McDowell and Dangl 2000; Kruidhof *et al.* 2012). Using standard methods (Babst *et al.* 2005), half of the plants randomly assigned to each of the three water availability treatments were designated as having the presence of pest pressure and treated with a solution of 0.125 % JA and 0.0625 % Triton X-100 surfactant (both purchased from Sigma-Aldrich Co. LLC, St Louis, MO, USA) in distilled water prior to the experimental period and then 2 days prior to the harvest period. Triton was added to the solution to improve the penetration of the JA through the waxy cuticles of tea leaves. Jasmonic acid was applied to the upper and lower surface of the newest leaf on each branch of tea plants designated with the presence of pest pressure. The plants designated with the absence of pest pressure were treated with a solution of 0.0625 % Triton surfactant in distilled water.

### Plant growth

Growth was measured by quantifying the number of new leaves and the height of tea plants during the experimental period.

### Sample collection

A sub-sample of 40 tea plants equally representing each of the water availability and pest pressure treatments was harvested by clipping three new leaves at their base using sharp shearing scissors. Samples were stored on ice and transferred to a lyophilizer (VirTis, SP Scientific) for a drying period of 48 h. Dry weights were recorded upon removal from the lyophilizer.

### Sample extraction

Leaf material was finely ground using a ball mill (Kleco pulverizer). Twenty milligrams of pulverized leaf material from each sample were extracted in 1.5 mL of 80 % aqueous HPLC-grade methanol (Fisher Scientific). The resulting mixture was vortexed for 30 s (Genie 2) and sonicated for 30 min at 20 °C (Quantrex 280, L&R Ultrasonics). Samples were centrifuged following sonication for 15 min at 15 000 rpm (Marathin Micro A, Fisher Scientific) and the supernatant was transferred to high-performance liquid chromatography (HPLC) vials for analyses of tea quality.

### Chemical analyses of tea functional quality

Tea quality was measured using HPLC to determine the concentration of eight antioxidant polyphenol compounds and three methylxanthine compounds linked to tea functional quality, including its health claims and stimulant properties. Individual methylxanthine compounds were aggregated into a measure of total methylxanthine concentrations (TMCs). In addition, total phenolic concentrations (TPCs) of tea leaves were measured. High-performance liquid chromatography was performed as previously described to measure antioxidant polyphenol and methylxanthine secondary metabolites (Unachukwu *et al.* 2010). The polyphenols measured include catechin (C), catechin gallate (CG), epicatechin 3-gallate (ECG), epigallocatechin (EGC), epigallocatechin 3-gallate (EGCG), gallic acid (GA) and gallocatechin 3-gallate (GCG; ChromaDex). The methylxanthines measured include caffeine, theobromine and theophylline (ChromaDex). A Waters 2695 (Milford, MA, USA) module equipped with a 996 photodiode array detector and a 4 μm, 250 × 4.6 mm ID, C-18 Synergi Fusion, reversed-phase column (Phenomenex, Torrance, CA, USA) was used for the HPLC analysis. Prior to the experimental run, the HPLC method was validated with respect to accuracy, precision, sensitivity and selectivity. For each sample, 5 μL were injected using a mobile phase of 0.05 % (v/v) trifluoroacetic acid in distilled water (Solvent A) and 0.05 % (v/v) trifluoroacetic acid in acetonitrile (Solvent B). The solvent gradient was set at a flow rate of 1 mL min^−1^ as follows: 12–21 % Solvent B from 0 to 25 min; 21–25 % Solvent B from 25 to 30 min. The column and autosampler temperatures were maintained at 38 and 4 °C, respectively. At the end of each run, the column was flushed with 100 % Solvent B for 10 min and was re-equilibrated for 5 min to starting conditions. Spectra were recorded from 254 to 400 nm and relevant peaks were detected at 280 nm on the basis of characteristic absorbance spectra and retention time. Analyte concentrations were determined using peak areas and the linearity determined by plotting signal versus concentration standard curve equations with the limit of detection and the limit of quantification in the ranges of 0.05–1 and 0.1–5 g mL^−1^, respectively.

Total phenolic concentration was determined spectrophotometrically using Folin–Ciocalteau reagent as previously described (Unachukwu *et al.* 2010). Samples were analysed in triplicate. Absorbance values were measured at 765 nm using a Benchmark Plus microplate spectrometer (Bio-Rad) and results expressed as gallic acid equivalents (GAE) in mg g^−1^ dry plant material. The concentration of polyphenols in tea samples was derived from a standard curve of GA concentration versus absorbance between 31.25 and 500 g mL^−1^.

### Statistical analysis

A fit model using a standard least squares means personality function and analysis of variance was performed using JMP 10.0 (SAS Institute Inc.) to determine how leaf growth and secondary metabolite concentrations vary among the precipitation and JA treatments. Data were analysed for the overall effect of water availability, JA treatment and their interactive effects. In addition, a multiple comparison using the least squares means Tukey's HSD method was applied to look at the difference between the three water availability treatments.

## Results

### Plant growth

**Figure 1.**
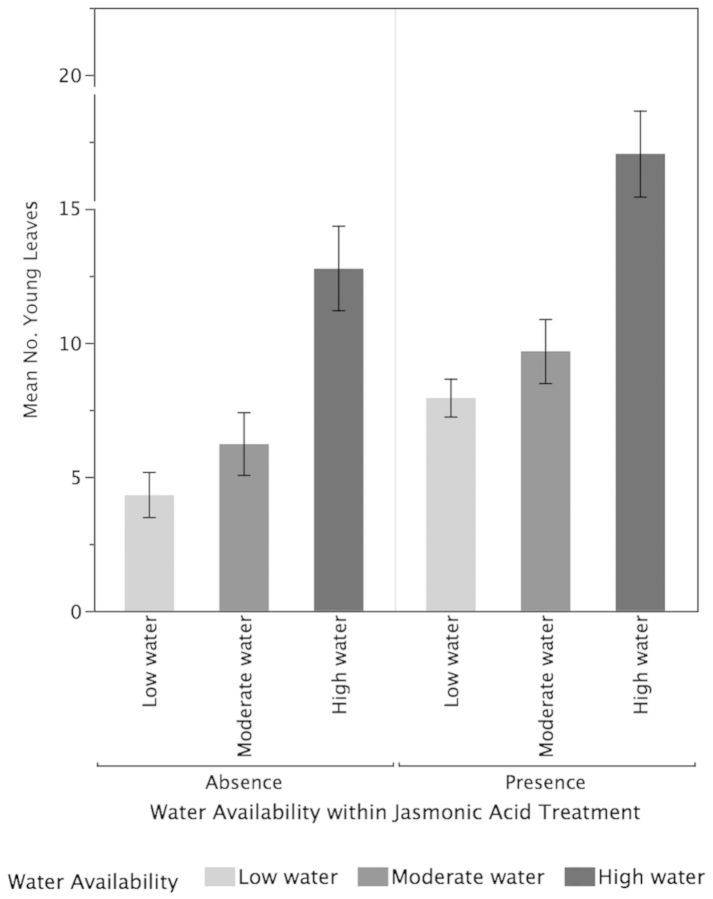
Effects of water availability and JA on leaf growth. Higher water availability (*P* < 0.001) and JA (*P* < 0.001) significantly increased the growth of new leaves while their interactive effect was not significant (*P* = 0.94). Values are means ± 1 standard error.

**Figure 2.**
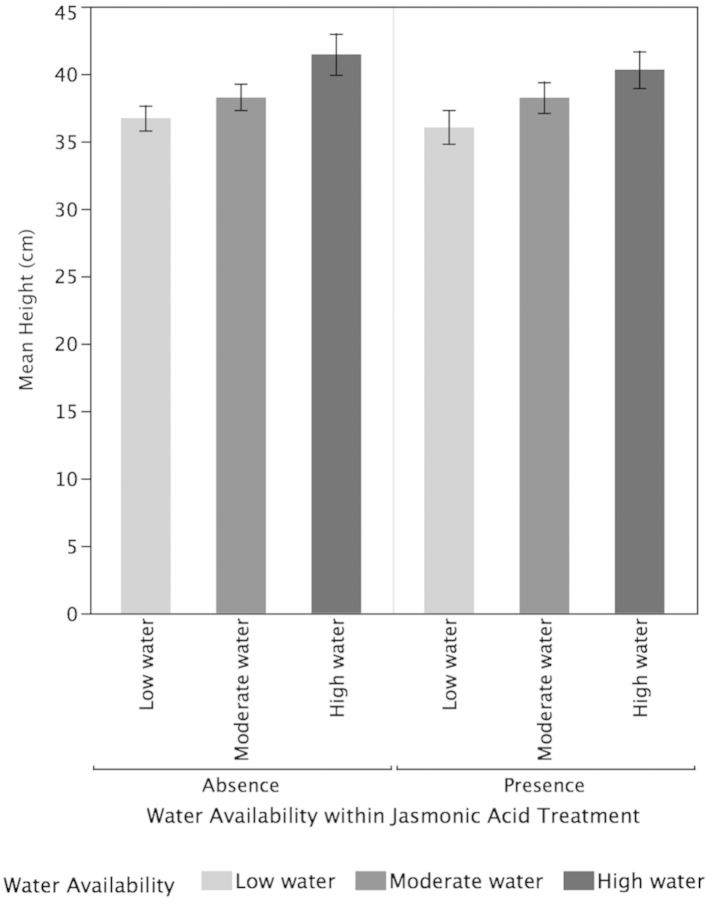
Effects of water availability and JA on plant height. Higher water availability (*P* = 0.001) but not JA (*P* = 0.54) or their interaction (*P* = 0.90) resulted in significantly increased plant height. Values are means ± 1 standard error.

Both higher water availability (*P* < 0.001) and JA (*P* < 0.001) significantly increased the growth of new leaves while their interactive effect was not significant (*P* = 0.94; Fig. 1). Overall, high-water plants had significantly more leaves than moderate-water plants (*P* < 0.0001) and low-water plants (*P* < 0.0001). The moderate-water plants and low-water plants did not differ significantly in the growth of new leaves (*P* = 0.24). Tea plants under the JA treatments had a significantly greater number of new leaves compared with plants that were not treated with JA (*P* = 0.0003). Higher water availability (*P* = 0.001) but not JA (*P* = 0.54) or their interaction (*P* = 0.90) resulted in significantly increased plant height (Fig. 2). The high–water-availability plants had significantly greater leaf growth than the low-water plants (*P* = 0.001) but did not differ significantly from the moderate-water-availability plants (0.059). While the low-water plants differed significantly in leaf growth from the high-water plants, they did not differ from the moderate-water plants (*P* = 0.22).

### Chemical analyses of tea functional quality

**Figure 3.**
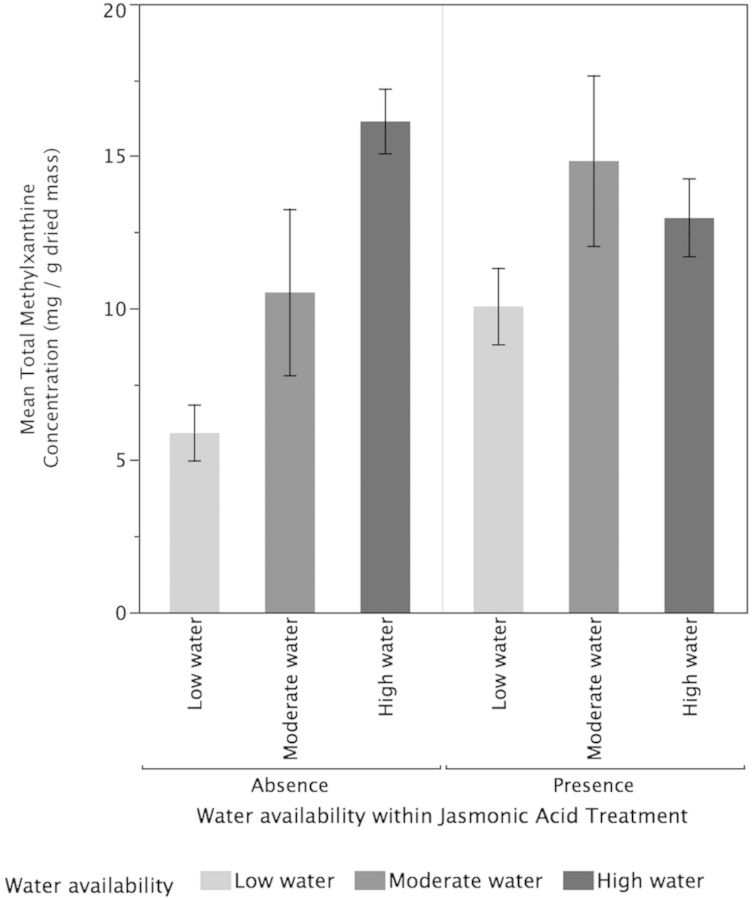
Effects of water availability and JA on TMC. Higher water availability (*P* < 0.001) significantly increased the TMCs of tea plants but there was no significant effect of JA treatments (*P* = 0.53) or the interaction between water and JA (*P* = 0.06). Values are means ± 1 standard error.

**Figure 4.**
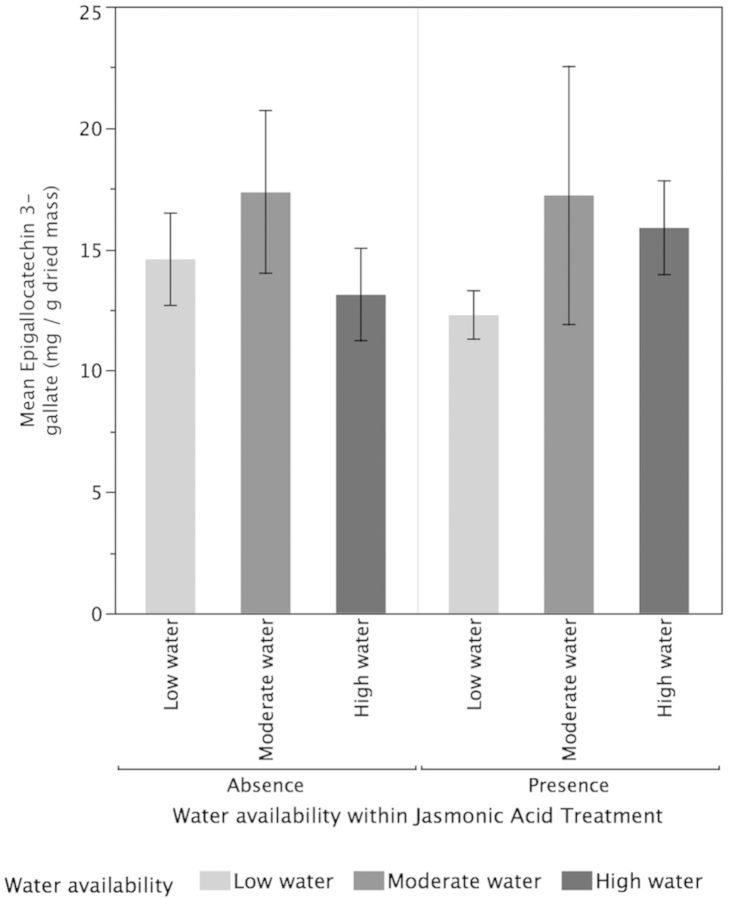
Effects of water availability and JA on the concentration of EGCG. Higher water availability (*P* = 0.37), JA treatments (*P* = 0.95) and their interactive effects had no significant effect on concentrations of EGCG. Values are means ± 1 standard error.

**Figure 5.**
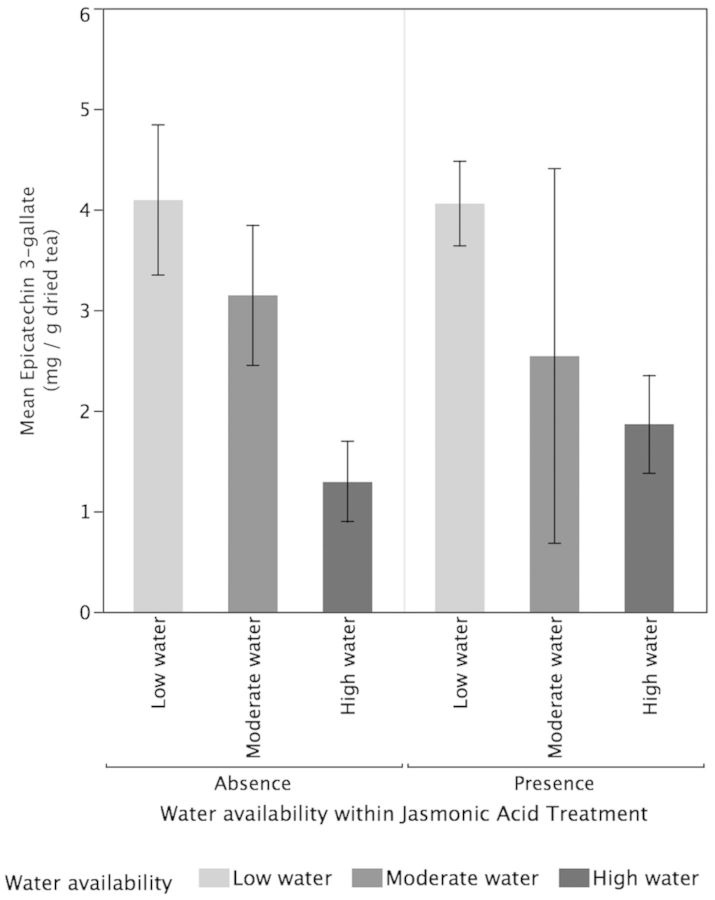
Effects of water availability and JA on the concentration of ECG. Higher water availability (*P* = 0.02) resulted in significantly lower ECG but the effect of JA (*P* = 0.982) and their interactive effects were not significant (*P* = 0.138). Values are means ± 1 standard error.

**Figure 6.**
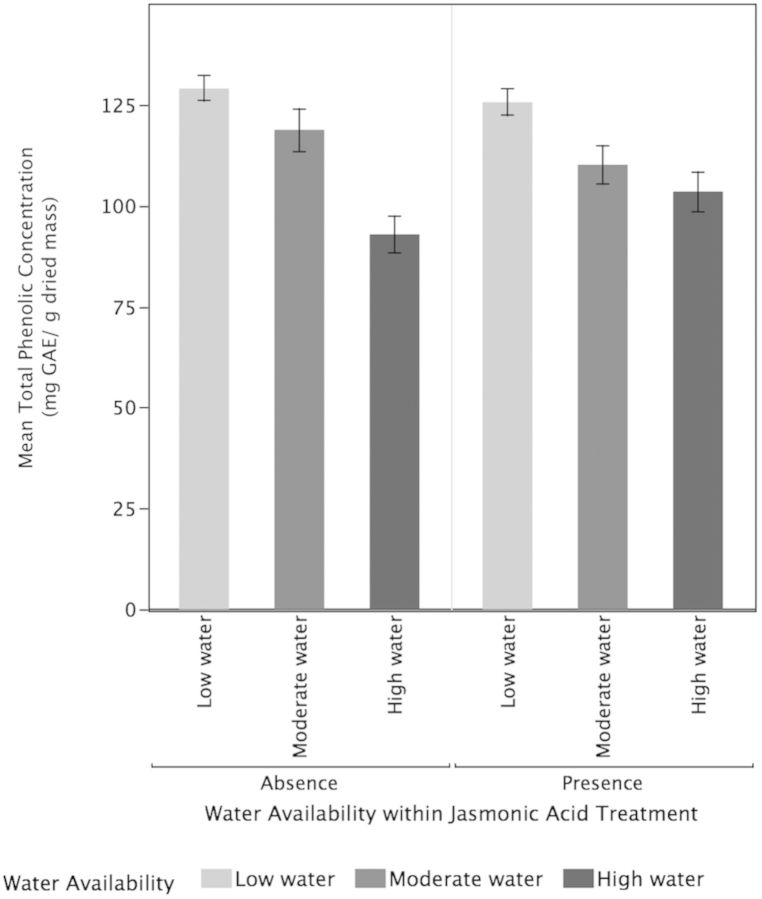
Effects of water availability and JA on TPC. Higher water availability (*P* < 0.0001) resulted in significantly higher TPC but there was no significant impact of JA (*P* = 0.89) and their interaction (0.09). Values are means ± 1 standard error.

Higher water availability (*P* < 0.001) significantly increased TMCs of tea plants but there was no significant effect of JA treatments (*P* = 0.53) or the interaction between water and JA (*P* = 0.06; Fig. 3). High-water plants (*P* < 0.0217) and moderate-water plants (*P* < 0.0009) had significantly higher concentrations of TMC compared with low-water plants but did not differ significantly from each other (*P* = 0.41). For the concentrations of EGCG, there was no significant effect for water availability (*P* = 0.37), JA treatments (*P* = 0.95) or their interactive effects (*P* = 0.68; Fig. 4). Neither the low-water (*P* = 0.28) nor the high-water (*P* = 0.49) treatments were significantly different from the moderate-water plants for EGCG concentrations. Additionally, there was no significant difference in EGCG concentrations between high- and low-water plants (*P* = 0.8891). In contrast, for ECG concentrations (Fig. 5), increased water availability (*P* = 0.02) resulted in significantly lower ECG but the effect of JA (*P* = 0.982) and the interactive effects of water and JA were not significant (*P* = 0.138). The high-water-availability treatments had significantly greater ECG concentrations compared with the low-water treatments (*P* = 0.0117) but did not differ significantly from the moderate-water treatments (*P* = 0.29). While the high- and low-water treatments differed significantly in their ECG concentrations, the moderate-water treatment did not differ significantly from either (*P* = 0.29). For TPC, higher water availability resulted in significantly higher TPC (*P* < 0.0001) but there was no significant impact of JA (*P* = 0.89) and their interaction (0.09; Fig. 6). High-water treatments had significantly greater TPC compared with moderate-water treatments (*P* = 0010) and low-water treatments (*P* < 0.0001). Moderate-water treatments had significantly higher TPC compared with low-water treatments (*P* = 0.0107).

## Discussion

This study supports the view that an increase in water availability results in a significant increase in growth of potted tea plants on the basis of both plant height and new leaves while the effects on secondary metabolites vary depending on chemical class. Higher water availability increased TMCs, decreased ECG levels and decreased TPCs of tea leaves. Epigallocatechin 3-gallate was the only tea functional quality parameter measured that was not significantly impacted by water availability treatments. Surprisingly, pest pressures as simulated by JA increased plant growth on the basis of new leaves, indicating that potted tea plants in a greenhouse setting may respond to pest pressures by prioritizing new leaf growth. Unexpectedly, JA had no significant effect on secondary metabolite chemistry. However, the interactive effects of water availability and simulated pest pressures show a trend to offset the direct effects of water availability on TMC and TPC. These findings point to the fascinating dynamics of climate change effects on tea plants with offsetting interactions within agro-ecosystems and the need for future climate studies to examine climate variables and pest pressures as well as their interactive effects.

In general, our findings concur with previous studies which found that altered water availability is a key driver of plant performance (Gulati and Ravindranath 1996; Dou *et al.* 2007) and significantly impacts both growth and secondary metabolite concentrations of tea plants (Gulati and Ravindranath 1996; Yao *et al.* 2005; Schepp 2009; Honow *et al.* 2010; CIAT 2011). Given the slow-growing nature of woody tea plants, the less notable effect of the treatments on plant height compared with leaves is expected. The reduced growth of plants under drought treatment in this study concurs with the widely accepted recognition that lower soil moisture content reduces photosynthesis, growth and survivability of plants (Kozlowski *et al.* 1991; Condit 1998). Shrubs with shallow roots, such as clonal tea shrubs, are particularly susceptible to drought effects and show severe water stress during the dry season (Tobin *et al.* 1997). Plants may respond to drought by closing their stomata to reduce water loss at the cost of eventually facing carbon starvation, or may keep their stomates open and face the risk of hydraulic failure (Zeppel *et al.* 2011).

The variability of the response of specific secondary metabolite concentrations to water variability emphasizes the complex changes in tea functional quality with forecasted climate change and concurs with studies showing idiosyncratic responses of individual compounds to environmental stress (Glynn *et al.* 2007). Caffeine is the primary secondary metabolite responsible for tea's stimulant properties and contributes to its bitter taste. Epicatechin 3-gallate and EGCG are prominent polyphenolic catechins in tea that contribute to tea's bitter taste as well as its sweet aftertaste, which is highly desirable. In addition, these compounds contribute to its antioxidant and anti-inflammatory properties and other medicinal attributes. Total phenolic concentration and antioxidant activity further contribute to the overall functional properties of tea. Consumers can discern changes in these compounds that influence their purchasing decisions (Ahmed *et al.* 2010). The methylxanthine caffeine is a nitrogen-based compound, while individual polyphenolic catechins along with the cumulative TPC measure represent carbon-based compounds.

We expected JA treatments to result in a large increase in these key secondary metabolites (Karban and Baldwin 1997; Kruidhof *et al.* 2012). Kruidhof *et al.* showed that proteinase inhibitors are highly induced by a second jasmonate, methyl jasmonate (124 % increase). Interestingly, they found that proteinase inhibitors were not expressed in glasshouse-grown plants. They suggest that the UV filtering properties prevent expression. Although they did not induce these glasshouse-grown plants with methyl jasmonate, it is possible that induction of many compounds is dependent on light quality. We suggest that future experiments should test the effects of jasmonates and pest pressures on tea plants grown in the field. Furthermore, this study used JA to simulate pest pressures that may provide an indication of what might happen when leaf-chewing caterpillars attack the plant. Tea is also attacked by leaf-sucking herbivores such as leaf hoppers, which induce different signalling pathways and thus may have very different effects on tea secondary chemistry and ultimately tea quality.

The significant impact of water availability on tea functional quality found in this study represents a conservative estimate of what would happen under field conditions, as manipulative studies are likely to underestimate plant responses to climate change for at least two reasons (Wolkovich *et al.* 2012). Field plants are exposed to many abiotic stressors (e.g. wind) that change plant chemistry and being older plants they typically have higher concentrations of many secondary metabolites. In addition, the interaction with additional climate variables, including temperature and carbon dioxide levels, would further exacerbate complexity with opposing or enhancing effects. In summary, future studies are needed that examine the interactive effects of multiple climatic factors with specialist tea pests in both controlled and field conditions.

## Conclusions

This study provides some of the first evidence on the multi-directionality of shifts in water availability, pest pressures and their interactive effects on tea quality. While numerous studies have documented the impact of climate change on crop yield, this study contributes to the knowledge gap on climate effects on crop quality that are crucial to examine for food security. Results indicate that while extreme drought and precipitation conditions might decrease or increase plant growth and functional quality, pest pressures may offset these effects. For example, drought conditions may result in a decline of both tea growth and stimulant properties of tea but pest pressures may offset these effects. If the changes in tea functional quality with water availability and herbivore pressures are indicative of broader climate change, tea production areas face increased heterogeneity with forecasted prolonged and more frequent droughts along with increased heavy precipitation events (Orlowsky and Seneviratne 2012; Seneviratne *et al.* 2012). Future research in both controlled and natural settings across spatial and temporal scales is needed to better understand the interplay between a range of climatic conditions, tea plants, herbivore pressures and other multi-trophic interactions.

## Sources of Funding

Our work was funded by a Tufts Collaborates Seed Grant, TEACRS Program at Tufts University (NIH National Institute of General Medical Sciences IRACDA-K12GM074869), National Science Foundation Research Experiences for Undergraduates Program (NSF DBI 1005082) and Tufts Institute for the Environment.

## Contributions by the Authors

All authors contributed to the overall study design. S.A. and T.G. designed water availability manipulations. S.A. and C.O. designed simulated pest pressure treatments. S.A. conducted greenhouse manipulations, coordinated study logistics and harvested samples. S.A., S.B. and U.U. were involved in the chemical and statistical analysis. A.S. helped with the experimental set-up of the pilot study. S.A. and C.O. primarily wrote the manuscript with contributions from all authors.

## Conflicts of Interest Statement

None declared.

## References

[PLT054C1] Ahmed S, Unachukwu U, Stepp JR, Peters CM, Long C, Kennelly E (2010). Pu-erh tea tasting in Yunnan, China: correlation of drinkers’ perceptions to phytochemistry. Journal of Ethnopharmacology.

[PLT054C2] Ahmed S, Peters CM, Long C, Meyer R, Unachukwu U, Litt A, Kennelly E, Stepp JR (2013). Biodiversity and phytochemical quality in indigenous and state-supported tea management systems of Yunnan, China. Conservation Letters.

[PLT054C5] Atkinson NJ, Urwin PE (2012). The interaction of plant biotic and abiotic stresses: from genes to the field. Journal of Experimental Botany.

[PLT054C100] Babst BA, Ferrieri RA, Gray DW, Lerdau M, Schlyer DJ, Schueller M, Thorpe MR, Orians CM (2005). Jasmonic acid induces rapid changes in carbon transport and partitioning in *Populus*. New Phytologist.

[PLT054C101] Berggren A, Bjorkman C, Bylund H, Ayres MP (2009). The distribution and abundance of animal populations in a climate of uncertainty. Oikos.

[PLT054C6] Boisvenue C, Running S (2006). Impacts of climate change on natural forest productivity—evidence since the middle of the 20th century. Global Change Biology.

[PLT054C7] Brenes-Arguedas T, Horton MW, Coley PD, Lokvam J, Waddell RA, Meizoso-O'Meara BE, Kursar TA (2006). Contrasting mechanisms of secondary metabolite accumulation during leaf development in two tropical tree species with different leaf expansion strategies. Oecologia.

[PLT054C8] Brenes-Arguedas T, Coley PD, Kursar TA (2009). Pests vs. drought as determinants of plant distribution along a tropical rainfall gradient. Ecology.

[PLT054C10] CIAT (2011). Future climate scenarios for Kenya's tea growing areas.

[PLT054C12] Coley P (1998). Possible effects of climate change on plant/herbivore interactions in moist tropical forests. Climatic Change.

[PLT054C13] Condit R (1998). Ecological implications of changes in drought patterns: shifts in forest composition in Panama. Climatic Change.

[PLT054C14] Dou J, Zhang Y, Yu G, Zhao S, Song Q (2007). Interannual and seasonal variations of energy and water vapor fluxes above a tropical seasonal rain forest in Xishuangbanna, SW China. Acta Ecologica Sinica.

[PLT054C16] Ewert F, Rounsevell MDA, Reginster I, Metzger MJ, Leemans R (2005). Future scenarios of European agricultural land use I. Estimating changes in crop productivity. Agriculture, Ecosystems and Environment.

[PLT054C102] Glynn C, Herms DA, Orians CM, Hansen RC, Larsson S (2007). Testing the growth-differentiation balance hypothesis: Dynamic responses of willows to nutrient availability. New Phytologist.

[PLT054C17] Gulati A, Ravindranath SD (1996). Seasonal variations in quality of Kangra tea (*Camellia sinensis* (L) 0 Kuntze) in Himachal Pradesh. Journal of the Science of Food and Agriculture.

[PLT054C18] Gutbrodt B, Mody K, Dorn S (2011). Drought changes plant chemistry and causes contrasting responses in lepidopteran herbivores. Oikos.

[PLT054C19] Gutbrodt B, Dorn S, Mody K (2012). Drought stress affects constitutive but not induced herbivore resistance in apple plants. Arthropod-Plant Interactions.

[PLT054C20] Herms DA, Mattson WJ (1992). The dilemma of plants—to grow or defend. The Quarterly Review of Biology.

[PLT054C21] Honow R, Gu KR, Hesse A, Siener R (2010). Oxalate content of green tea of different origin, quality, preparation and time of harvest. Urological Research.

[PLT054C22] Parry M, Canziani O, Palutikof J, Van Der Linden P, Hanson C, IPCC (2007). Climate change 2007: impacts, adaptation and vulnerability. Contribution of Working Group II to the fourth assessment report of the Intergovernmental Panel on Climate Change.

[PLT054C23] Jamieson MA, Trowbridge AM, Raffa KF, Lindroth RL (2012). Consequences of climate warming and altered precipitation patterns for plant–insect and multitrophic interactions. Plant Physiology.

[PLT054C24] Karban R, Baldwin IT (1997). Induced responses to herbivory.

[PLT054C25] Kozlowski TT, Kramer PJ, Pallardy SG (1991). The physiological ecology of woody plants.

[PLT054C26] Kruidhof HM, Allison JD, Hare D (2012). Abiotic induction affects the costs and benefits of inducible herbivore defenses in *Datura wrightii*. Journal of Chemical Ecology.

[PLT054C28] Lin Y, Tsai Y, Tsay J, Lin J (2003). Factors affecting the levels of tea polyphenols and caffeine in tea leaves. Journal of Agriculture and Food Chemistry.

[PLT054C29] Lobell DB, Schlenker W, Costa-Roberts J (2011). Climate trends and global crop production since 1980. Science.

[PLT054C30] Maplecroft (2011). www.maplecroft.com.

[PLT054C31] McDowell JM, Dangl JL (2000). Signal transduction in the plant immune response. Trends in Biochemical Sciences.

[PLT054C33] Nelson GC, Rosegrant MW, Koo J, Robertson R, Sulser T, Zhu T, Ringler C, Msangi S, Palazzo A, Batka M, Magalhaes M, Valmonte-Santos R, Ewing M, Lee D (2009). Climate change: impact on agriculture and costs of adaptation.

[PLT054C34] Orlowsky B, Seneviratne S (2012). Global changes in extreme events: regional and seasonal dimension. Climatic Change.

[PLT054C36] Porter JR, Semenov MA (2005). Crop responses to climatic variation. Philosophical Transactions of the Royal Society B.

[PLT054C38] Schepp K (2009). Strategy to adapt to climate change for Michimikuru tea farmers in Kenya.

[PLT054C39] Schlenker W, Lobell DB (2010). Robust negative impacts of climate change on African agriculture. Environmental Research Letters.

[PLT054C40] Seneviratne S, Nicholls N, Easterling D, Goodess C, Kanae S, Kossin J, Luo Y, Marengo J, McInnes K, Rahimi M, Reichstein M, Sorteberg A, Vera C, Zhang X, Field CB, Barros V, Stocker TF, Qin D, Dokken DJ, Ebi KL, Mastrandrea MD, Mach KJ, Plattner G-K, Allen SK, Tignor M, Midgley PM (2012). Changes in climate extremes and their impacts on the natural physical environment. Managing the risks of extreme events and disasters to advance climate change adaptation. A special report of Working Groups I and II of the Intergovernmental Panel on Climate Change (IPCC).

[PLT054C103] Tharayil N, Suseela V, Triebwasser DJ, Preston CM, Gerartd PD, Dukes JS (2011). Changes in the structural composition and reactivity of *Acer rubrum* leaf litter tannins exposed to warming and altered precipitations: climatic stress-induced tannins are more reactive. New Phytologist.

[PLT054C41] Tobin MF, Lopez OR, Kursar TA (1997). Drought response of tropical understory species with long and short leaf lifespans. Biotropica.

[PLT054C42] Unachukwu U, Ahmed S, Kavalier A, Lyles J, Kennelly E (2010). Variation of phenolic and methylxanthine composition and anti-oxidant activity among white and green teas (*Camellia sinensis* var. *sinensis* (L.) Kuntze Theaceae). Journal of Food Science.

[PLT054C43] Wolkovich EM, Cook BI, Allen JM, Crimmins TM, Betancourt JL, Travers SE, Pau S, Regetz J, Davies TJ, Kraft NJB, Ault TR, Bolmgren K, Mazer SJ, McCabe GJ, McGill BJ, Parmesan C, Salamin N, Schwartz MD, Cleland EE (2012). Warming experiments underpredict plant phenological responses to climate change. Nature.

[PLT054C44] Yao L, Caffin N, D'Arcy B, Jiang Y, Shi J, Singanusong R, Liu X, Datta N, Kakuda Y, Xu Y (2005). Seasonal variations of phenolic compounds in Australia-grown tea (*Camellia sinensis*). Journal of Agriculture and Food Chemistry.

[PLT054C45] Zeppel MJB, Adams HD, Anderegg WRL (2011). Mechanistic causes of tree drought mortality: recent results, unresolved questions and future research needs. New Phytologist.

